# Ultrafast Return to Sport in International Feminine Football Following Arthroscopic Anatomic Lateral Ankle Ligament Reconstruction Using Gracilis Autograft: A Case Report

**DOI:** 10.1155/cro/5568199

**Published:** 2026-07-20

**Authors:** Pierre-Jean Lambrey, Germain Saniel, Ronny Lopes

**Affiliations:** ^1^ Department of Orthopaedic and Trauma Surgery, Lille University Hospital, Lille, France, chru-lille.fr; ^2^ Performance Department, Olympique Lyonnais, Decines-Charpieu, France; ^3^ Department of Orthopaedic Surgery and Sports Medicine, Centre Orthopedique Santy and Hopital Prive Jean Mermoz, Lyon, France

**Keywords:** ankle instability, arthroscopic ligament reconstruction, football, gracilis autograft, high-performance rehabilitation, return to sport

## Abstract

Lateral ankle instability is a common condition in elite football, but returning to play following arthroscopic anatomic ligament reconstruction (AALR) typically requires 4–6 months. We report the case of a 24‐year‐old professional female football player who achieved a full return to elite competition and national team duties within 12 weeks following arthroscopic ALR of the anterior talofibular (ATFL) and calcaneofibular (CFL) ligaments using an autologous gracilis graft. Preoperative instability followed recurrent sprains incurred during the World Cup, with examination and imaging demonstrating complete lateral ligament complex insufficiency. Arthroscopic exploration identified a complete ATFL/CFL rupture, anterolateral impingement, and a lateral talar chondral rail lesion, which were treated by synovectomy, debridement, and AALR. Rehabilitation followed an intensive, criterion‐based protocol integrating immediate full weight‐bearing, progressive range of motion, neuromuscular training, strength symmetry targets, and field‐based functional testing. At 12 weeks, the athlete achieved full functional symmetry: AnkleGO score 25/25, FAAM‐ADL and FAAM‐Sport 100%, ALR‐RSI 100%, symmetric hop performance, and unrestricted football‐specific metrics. She returned to international duty without restrictions and completed full match participation. This case illustrates that arthroscopic anatomic reconstruction combined with structured high‐performance rehabilitation can enable ultrafast return to elite football, challenging conventional timelines while illustrating an accelerated, criterion‐based return in a single elite athlete rather than a generalizable target.

## 1. Introduction

Lateral ankle sprains are among the most common injuries in sport, accounting for more than 25% of all sports‐related injuries [1], and are particularly frequent in elite football [2, 3], where they are a leading cause of time lost and long‐term instability [4]. Professional football exposes athletes to rapid changes of direction, high‐velocity decelerations, and repeated contact situations, all of which increase the risk of lateral ligament complex failure. Athletes who sustain an acute sprain develop chronic lateral ankle instability (CLAI), characterized by mechanical laxity, proprioceptive deficits, recurrent “giving‐way,” and performance limitations [5]. When conservative treatment fails despite neuromuscular rehabilitation, taping, orthotic correction, and sport‐specific conditioning, surgical stabilization becomes necessary to restore functional performance. Broström‐type procedures [6–8] are widely used but may be insufficient in cases of complete ligament insufficiency, high‐demand athletes, or chronic tissue degeneration. In such situations, anatomic ligament reconstruction using tendon grafts offers mechanical resistance and reliable restoration of the ATFL/CFL complex, especially when both ligaments are compromised. Although meta‐analytic evidence suggests that this technique may be associated with longer return‐to‐sport (RTS) timelines compared with direct repairs, it also indicates improved midterm functional stability [9, 10].

Despite improved surgical techniques, RTS after anatomic ligament reconstruction is generally achieved at a mean of approximately 7.5 months in athletic populations, and fewer than half of competitive athletes return to their preinjury level in large series [8, 9, 11–15]. Compared with Broström–Gould repair, anatomical reconstruction of the ligament is associated with lower recurrence rates [16], although it is generally followed by a longer recovery period and return to play (RTP). However, performing the procedure arthroscopically may help reduce surgical morbidity and facilitate postoperative recovery. Evidence regarding accelerated return after anatomic reconstruction remains scarce, and even more so in elite women′s football, where scientific literature is particularly limited. Achieving an earlier yet safe RTS requires robust mechanical stabilisation but also an individualised, criterion‐based reathletisation program integrating strength, neuromuscular control, sport‐specific mechanical loads, psychological readiness, and objective performance testing [17–19].

This paper describes the case of a 24‐year‐old professional footballer, a member of the French national team, who was able to fully resume international competition within 12 weeks following arthroscopic anatomical reconstruction of the ligament using an autograft from the gracilis muscle. We present the surgical findings, postoperative course, and detailed sports rehabilitation program, supported by objective functional and performance measurements from the rehabilitation records.

## 2. Patient Case

The patient was a 24‐year‐old professional left‐back (1.61 m, 58.5 kg) competing at the highest international level with the French national team. The patient gave her consent for this study, and we obtained approval from the institutional review board with the number: COS‐RGDS‐2025‐10‐002‐LOPES‐R.

She presented with persistent left ankle instability following two severe sprains sustained during the World Cup, on a background of multiple previous sprains and a clinically documented second‐degree flexible flatfoot. Despite comprehensive high‐performance conservative management—including proprioceptive retraining, neuromuscular conditioning, and customized orthotic correction—she continued to experience mechanical giving‐way and functional apprehension during football‐specific actions such as cutting, acceleration, and aerial landings. Preoperative patient‐reported outcomes confirmed predominantly mechanical instability with relatively preserved self‐reported function: FAAM‐ADL 100%, FAAM‐Sport 90%, Ankle‐GO 20/25, and ALR‐RSI 91%. Clinical examination demonstrated marked frontal‐plane laxity with positive anterior drawer and talar tilt responses, consistent with lateral ligament complex insufficiency. MRI confirmed a severe lateral ligament injury, with rupture and dilaceration of the proximal (fibular) insertion of the ATFL and rupture–dilaceration of the CFL with distal predominance. Associated findings included talocrural and subtalar joint effusion and a small Chopart joint effusion, with intact distal tibiofibular syndesmotic ligaments. Stress radiographs were not performed; the diagnosis of mechanical lateral instability relied on clinical examination and MRI.

Given the persistence of symptoms despite maximal conservative treatment, combined with documented mechanical instability and the elite performance requirements of the athlete′s position, surgical reconstruction of the lateral ligament complex was indicated.

### 2.1. Surgical Technique

**Figure 1:**
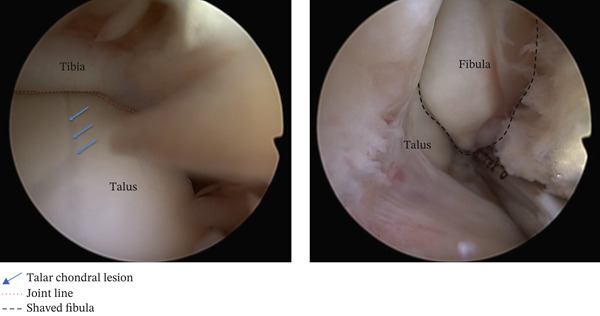
Initial arthroscopic inspection revealing a shaved fibular footprint consistent with complete ligament insufficiency along with an associated chondral lesion of the talus.

**Figure 2:**
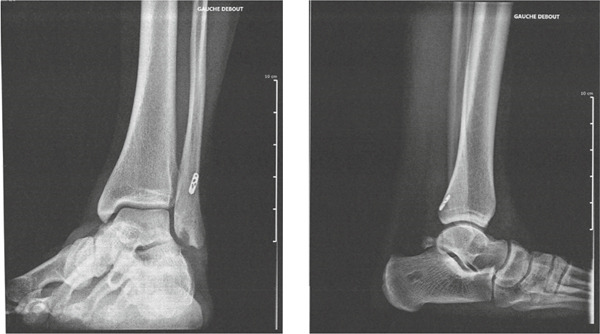
Postoperative x‐rays.

Surgery was performed under general anesthesia with tourniquet control according to the operative report. A gracilis tendon autograft was harvested through an anteromedial approach and prepared to a diameter of 4 mm using FiberLoop sutures. Standard anteromedial arthroscopic access allowed full intra‐articular assessment, revealing a complete Stage 4 injury of the ATFL, absence of CFL visualization consistent with complete ligament insufficiency, an anterolateral soft‐tissue impingement, and a characteristic lateral talar chondral “rail” lesion (Figure 1). No syndesmotic or deltoid ligament abnormalities were identified. Arthrolysis, synovectomy, and meticulous debridement of the ATFL and CFL footprints were performed to prepare the reconstruction footprint. A fully arthroscopic anatomic reconstruction of both ATFL and CFL was then carried out using the gracilis autograft [20]: a 5.0‐mm talar tunnel was created, the fibular tunnel was prepared for ACL TightRope fixation, and a 5.5‐mm calcaneal tunnel was drilled. The graft was secured using a 4.75‐mm talar biotenodesis screw and a 5.5‐mm calcaneal screw, recreating the native trajectories of the ATFL and CFL. Fixation was performed with the ankle maintained at 90° of neutral alignment. The final testing demonstrated excellent tension and anatomic restoration of the lateral ligament complex. Figure 2 is showing x‐ray postoperative assessment.

### 2.2. Postoperative Protocol (Table 1, Figure 3)

**Table 1:** Rehabilitation and return‐to‐sport timeline following arthroscopic anatomic ATFL/CFL reconstruction.

Phase	Timeframe	Main objectives	Key interventions	Progression criteria
Phase 1	Weeks 0–2	Graft protection, pain and edema control, early neuromuscular activation	Walking boot full time; protected weight‐bearing; cryotherapy; elevation; scar protection; isometric activation of calf and intrinsic foot muscles	Pain VAS 0–2; no wound complication; tolerance to protected loading
Phase 2	Weeks 2–4	Restore ankle mobility and neuromuscular control	Progressive dorsiflexion and plantarflexion ROM; lymphatic drainage; scar mobilization; peroneal and intrinsic foot activation; balance on stable surfaces	Weight‐bearing lunge test > 12 cm, progressing toward < 1.5 cm side‐to‐side difference; reduced effusion; stable single‐leg stance
Phase 3	Weeks 4–8	Strength restoration and proprioceptive progression	Concentric and eccentric strengthening of calf and peroneals; hamstring strengthening (gracilis harvest); hip and pelvic stabilization; unstable‐surface balance; perturbation training	Calf isometric strength 1.5–2 × body weight with < 10% interlimb asymmetry; single‐leg heel‐rise > 27 repetitions (< 10%); hamstring and adductor asymmetry within thresholds
Phase 4	Weeks 8–10	Plyometric exposure and running reintroduction	Bilateral to unilateral hops; landing‐mechanics training; walk–run progression; linear accelerations; controlled decelerations	Surgeon‐validated running (antigravity treadmill from Day 34); single‐leg hop symmetry index > 0.80; no stiff‐landing pattern; no pain or effusion
Phase 5	Weeks 10–12	Field‐based reathletization and football‐specific loading	Change‐of‐direction drills; agility sequences; football‐specific movement patterns; advanced plyometrics (CMJ, DJ, SLCMJ); GPS‐monitored workloads	CMAS‐guided change of direction; plyometric progression toward return‐to‐train thresholds (CMJ RSI‐mod > 0.5, DJ RSI > 1.5); hop symmetry index > 0.90; progressive GPS‐monitored workload
Phase 6	Week 12	Objective return‐to‐train assessment	Ankle‐GO battery, FAAM‐ADL, FAAM‐Sport, ALR‐RSI, hop tests, SEBT, strength and plyometric testing	Ankle‐specific criteria met (Ankle‐GO 25/25; FAAM‐ADL and FAAM‐Sport 100%; ALR‐RSI 100%; symmetric single‐leg hop); residual performance deficits (calf isometric symmetry, dorsiflexion ROM, plyometric reactivity) addressed thereafter
Return to sport	≥ Week 12	Full reintegration	Unrestricted team training and competitive match play	No pain or instability; ankle‐specific return‐to‐sport criteria fulfilled

Abbreviations: ALR‐RSI, Ankle Ligament Reconstruction—Return to Sport after Injury scale; BW, body weight; CMAS, Cutting Movement Assessment Score; CMJ, countermovement jump; COD, change of direction; DJ, drop jump; FAAM‐ADL, Foot and Ankle Ability Measure—Activities of Daily Living; FAAM‐Sport, Foot and Ankle Ability Measure—Sport subscale; GPS, global positioning system; ROM, range of motion; RSI, reactive strength index; RTP, return to play; RTT, return to train; SEBT, Star Excursion Balance Test; SLCMJ, single‐leg countermovement jump; SLDJ, single‐leg drop jump; VAS, visual analogue scale.

**Figure 3:**
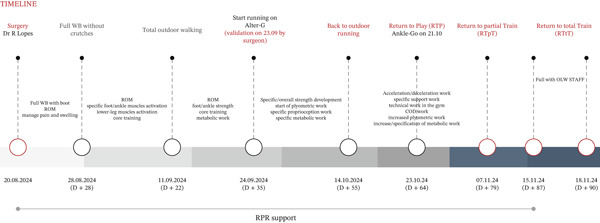
Physiotherapy/performance timeline.

Rehabilitation followed a strictly criterion‐based, performance‐oriented, and load‐monitored progression designed by the medical staff of the team and the surgeon to restore not only ankle stability but also full football‐specific performance capacity.

The phase structure, immediate protected weight‐bearing, criterion‐based progression, and the Ankle‐GO RTS battery constituted our institution′s standard postoperative pathway. The element individualized for this athlete was the pace of progression, which was governed by the serial normalization of the Ankle‐GO score and RTS functional tests rather than by a fixed time‐based schedule.

During the first two postoperative weeks, immediate protected weight‐bearing in a walking boot was allowed, whereas rehabilitation focused on pain modulation, edema control, scar management, and preservation of neuromuscular activation of the lower limb without exposing the graft to inversion stress. From Week 2, physiotherapy emphasized progressive restoration of sagittal‐plane ankle mobility, lymphatic drainage, scar mobilization, and early neuromuscular reactivation of the intrinsic foot muscles, peroneal group, and calf complex. Between Weeks 4 and 8, loading strategies were gradually intensified through isometric, concentric, and eccentric strengthening of the plantar flexors and evertors, combined with targeted hamstring strengthening addressing gracilis harvest, and proximal hip and trunk stabilization to optimize lower‐limb kinetic chain control. Proprioceptive training progressed from stable to unstable surfaces, incorporating perturbation‐based tasks and dynamic single‐leg alignment control. Plyometric exposure was introduced in a graded manner, starting with bilateral pogo jumps and progressing toward unilateral hops, with continuous monitoring of landing symmetry, stiffness regulation, and force absorption capacity. Running was reintroduced on an antigravity treadmill after surgeon validation (Day 34) before outdoor running. From Week 8 onward, rehabilitation transitioned into field‐based reathletization, integrating progressive running volumes, linear accelerations, and controlled decelerations, followed by sport‐specific change‐of‐direction drills with increasing angles, velocities, and cognitive demands. Plyometric performance was further developed using countermovement jumps, drop jumps, and single‐leg reactive tasks to restore reactive strength index and stretch‐shortening cycle efficiency. Throughout the process, objective neuromuscular, strength, balance, and plyometric metrics were systematically assessed to guide progression and prevent asymmetry‐driven overload. At 12 weeks postoperatively, the athlete demonstrated full restoration of functional and performance criteria, including perfect AnkleGO score [18], maximal FAAM‐ADL and FAAM‐Sport scores [21], complete psychological readiness on the ALR‐RSI scale [19], symmetric hop and balance performance, normalized isometric and endurance strength of the calf complex, and restored plyometric reactivity. These objective findings supported clearance for unrestricted return to team training and competitive match play, allowing the athlete to reintegrate professional and international football activities without pain, instability, or performance limitation.

In an elite athlete, these patient‐reported scores are subject to a ceiling effect and remained high despite disabling mechanical instability (recurrent giving‐way, positive anterior drawer and talar tilt, and complete ATFL/CFL insufficiency); the surgical objective was therefore the restoration of mechanical stability rather than the correction of a collapsed functional score.

### 2.3. Return to Sport

The athlete resumed on‐field progressive work at Day 59, then reintegrated elite‐level team training. Progression between phases was governed by predefined quantitative criteria (Table 2). At the 12‐week return‐to‐train assessment, the athlete met all ankle‐specific functional and psychological criteria—Ankle‐GO 25/25, FAAM‐ADL and FAAM‐Sport 100%, ALR‐RSI 100%, symmetric single‐leg hop performance, and normal balance and single‐leg stance—whereas some performance parameters (calf isometric symmetry, dorsiflexion range of motion, and plyometric reactivity) were not yet fully normalized and continued to be progressed after return to training (Table 3). Following completion of return‐to‐train criteria, the athlete was reintegrated into full team training. She was named in the matchday squad for a European club competition fixture at 92 days postoperatively (unused substitute) and made her first competitive appearance in a national league fixture at 95 days postoperatively, corresponding to approximately 13.5 weeks after surgery. This report has several limitations. First, it describes a single elite athlete managed within a specialized multidisciplinary setting, which limits generalizability. Second, although clinical follow‐up at approximately 18 months postoperatively revealed no recurrence of instability, no giving‐way, and no ankle‐related pain or functional limitation, with continued competitive play, longer‐term data are needed to confirm the durability of the result. Third, no postoperative imaging of graft integration was performed, as the uneventful clinical course did not prompt such investigation; consequently, structural graft maturation was not objectively documented. Finally, some performance parameters (calf isometric strength symmetry, dorsiflexion range of motion, and plyometric reactivity) had not fully normalized at return to train and continued to be addressed thereafter. These limitations should be considered when interpreting the rapid return‐to‐sport timeline reported here.

**Table 2:** Test battery and quantitative thresholds used to guide rehabilitation progression and return‐to‐sport decision‐making.

Domain	Test	Threshold/cut‐off	Symmetry criterion
Dorsiflexion ROM	Weight‐bearing lunge test	> 12 cm	Side‐to‐side difference < 1.5 cm
Calf strength	Isometric soleus (5 s)	1.5–2 × *b* *o* *d* *y* *w* *e* *i* *g* *h* *t*	Interlimb asymmetry < 10%
Calf endurance	Single‐leg heel‐rise	> 27 repetitions	< 10%
Hamstring strength	Nordic hamstring	≥ 260 N (4 × BW + 26.1 N)	< 15%
Adductors	Bridge 60°/squeeze 0°	>3 N/kg/> 2 N/kg	< 10%
Plyometric (bilateral)	CMJ RSI‐mod; drop‐jump RSI	CMJ: RTP > 0.4/RTT > 0.5; DJ: RTP > 1.3/RTT > 1.5	—
Plyometric (unilateral)	SLCMJ; SLDJ	SLCMJ eccentric velocity > 0.7; SLDJ RSI: RTP > 0.5/RTT > 1.5	—
Hop	Single‐leg hop for distance	No stiff‐landing pattern	Symmetry index RTP > 0.80/RTT > 0.90
Change of direction	Cutting Movement Assessment Score (CMAS)	Objective score < 3	—
Ankle‐specific RTS	Ankle‐GO (FAAM‐ADL, FAAM‐Sport, ALR‐RSI, Figure‐of‐8, side hop, single‐leg stance, SEBT)	Composite score/25	—

*Note:* Threshold values served as guidance—they are partly derived from male‐athlete reference data—and were interpreted alongside the linear progression of each parameter and validated by the operating surgeon.

Abbreviations: BW, body weight; CMJ, countermovement jump; RSI, reactive strength index; RTP, return to play; RTT, return to train; SEBT, Star Excursion Balance Test; SLCMJ, single‐leg countermovement jump; SLDJ, single‐leg drop jump.

**Table 3:** Return‐to‐train assessment at 12 weeks (Day 85): operated versus contralateral limb.

Domain	Test (metric)	Operated limb (left)	Contralateral limb (right)	Threshold/symmetry criterion
Ankle‐specific readiness	Ankle‐GO composite score (/25)	25	—	Maximal score
	FAAM‐ADL/FAAM‐Sport (%)	100/100	100/100	—
	ALR‐RSI (%)	100	—	—
Functional hop tests	side hop test (s)	6.61	6.67	—
	Figure‐of‐8 test (s)	9.24	8.98	—
Single‐leg jump	Single‐leg hop‐for‐distance symmetry index	1.0	—	RTT > 0.90; no stiff landing
	SLCMJ height (cm)	13.1	13.9	Norm 17.4 ± 3.4
	SLDJ reactive strength index	0.64	0.80	RTT > 1.5
Dynamic balance (SEBT)	Anterior reach (cm)	58.3	57.7	—
	Posteromedial reach (cm)	113.0	115.3	—
	Posterolateral reach (cm)	122.3	118.7	—
	single‐leg stance (errors)	0	1–3	—
Calf strength	Isometric soleus, 5 s (N)	731	841	Asymmetry 13% (criterion < 10%)
	Single‐leg heel‐rise (repetitions)	43	45	Asymmetry 5% (criterion < 10%)
Ankle mobility	Weight‐bearing lunge test (cm)	10	13	> 12 cm; side‐to‐side < 1.5 cm

*Note:* Values obtained at the return‐to‐train screening (Day 85, operated left ankle) and concurrent Ankle‐GO functional testing. Lower times indicate better performance for the side hop and Figure‐of‐8 tests. Ankle‐specific functional symmetry and psychological readiness were fully restored, whereas calf isometric strength symmetry and dorsiflexion range of motion had not yet normalized and were addressed in continued training.

Abbreviations: ALR‐RSI, Ankle Ligament Reconstruction—Return to Sport after Injury scale; FAAM‐ADL, Foot and Ankle Ability Measure—Activities of Daily Living; FAAM‐Sport, Foot and Ankle Ability Measure—Sport subscale; RTT, return to train; SEBT, Star Excursion Balance Test; SLCMJ, single‐leg countermovement jump; SLDJ, single‐leg drop jump.

## 3. Discussion

CLAI represents a major concern in elite football players, given its impact on performance, recurrence risk, and long‐term joint health. Although most ankle sprains heal with conservative treatment, up to 40% of patients may develop persistent mechanical or functional instability requiring surgical management [18, 22]. In professional athletes, surgical stabilization aims not only to restore mechanical stability but also to optimize RTS quality, confidence, and durability [14].

Anatomic lateral ankle stabilization has progressively replaced nonanatomic techniques, as it better restores native ligament biomechanics and reduces the risk of degenerative changes [22]. Early ligament repair in elite athletes may allow fast RTS when performed in acute Grade III injuries. Hong and Calder [8] reported a median RTS time of 69 days following early Broström repair, with excellent RTS rates. However, associated lesions, particularly osteochondral talar lesions, significantly delayed RTS, reporting the importance of treating intra‐articular pathologies.

When ligament remnants are insufficient, anatomic reconstruction using tendon grafts is recommended, particularly in cases of generalised laxity, revision surgery, or high‐demand athletes [16]. Comparative data suggest that ligament reconstruction is associated with superior midterm functional outcomes compared with arthroscopic Broström repair, despite delayed RTS [16]. Feng et al. [10] demonstrated better AOFAS and Karlsson scores at 1–2 years after reconstruction, although RTS occurred later than after repair. These results corroborate the idea that reconstruction offers greater long‐term mechanical reliability at the cost of a longer healing period.

To address these issues, arthroscopic approaches to lateral ankle stabilisation appear to facilitate postoperative recovery by minimising soft tissue disruption compared with open techniques [22–25]. By avoiding extensive dissection of the retinaculum and surrounding periarticular structures, arthroscopy reduces surgical morbidity. This minimally invasive strategy can therefore contribute to faster functional recovery and better short‐term outcomes, particularly in high‐level athletes, as shown in the randomized controlled trial of Su et al. [26] about 57 CLAI treated by anatomical ligament reconstruction. The arthroscopic all‐inside technique was associated with faster postoperative recovery, as evidenced by earlier return to full weightbearing (8.9 vs. 11.7 weeks), jogging (17.9 vs. 20.9 weeks), and recreational sports (22.4 vs. 26.5 weeks), as well as lower pain levels and superior functional scores during the first 3–6 postoperative months. The use of arthroscopic approaches also allows precise graft placement and concomitant lesion management [8, 14].

Recent cohort studies confirm high RTS rates after arthroscopic anatomic reconstruction, with 85%–92% of athletes resuming sport and approximately 75%–80% returning to their preinjury level [14, 17, 22]. The systematic review and meta‐analysis by Li et al. [22] reported that 95% of patients returned to some level of sport and 83% returned to their preinjury level after anatomic lateral ankle stabilization, with a mean RTS time of 12.4 weeks. It also reports that elite athletes demonstrated higher RTS rates than recreational populations. Importantly, age and body mass index negatively influenced RTS probability. Psychological readiness plays a crucial role, and ALR‐RSI scores have been shown to outperform functional scores in predicting RTS at the preinjury level [17]. Moreover, Hardy et al. [18] demonstrated that the Ankle‐GO score provides a validated tool for RTS decision‐making after lateral ankle reconstruction.

In this case, the patient presented with chronic instability associated with both ATFL and CFL insufficiency and a talar chondral lesion, making simple repair unsuitable. In such conditions, anatomic reconstruction remains the most reliable option to restore multiplanar stability and protect long‐term joint function. It illustrates an exceptionally rapid and high‐level RTS, with a return to international competition at 3 months postoperatively, despite the presence of an associated intra‐articular lesion and the use of an anatomic ligament reconstruction rather than a direct repair. This ultrafast recovery likely reflects several favorable factors: elite neuromuscular conditioning, strict rehabilitation adherence, multidisciplinary monitoring, objective functional testing, and psychological readiness. In this elite context, the small numerical gain in already near‐maximal functional scores is clinically meaningful, corresponding to the difference between persistent mechanical apprehension and unrestricted return to the highest competitive level.

Return to competitive play at approximately 13.5 weeks represents roughly half the mean time to return to sport reported after the same procedure in a comparable athletic cohort (7.5 months) [13]; as a single case report, this is not intended to define a generalizable benchmark but to document that such an accelerated timeline can be achieved within a dedicated multidisciplinary structure.

No postoperative imaging of graft integration was obtained in this case; however, the rationale for awaiting imaging confirmation of ligamentization before return to sport is not well established. In arthroscopic anatomic ATFL/CFL reconstruction with a gracilis autograft, ligamentization is a real but prolonged process; graft MRI signal decreases progressively from 6 to 24 months [27] and SNQA values decline significantly up to 12 months [28], whereas biomechanical assessment shows the reconstructed graft to be initially stiffer than the native ATFL, evolving during maturation [29]. Mechanical stability is therefore restored early, before biological maturation is complete, and full ligamentization continues for 1–2 years—far beyond any realistic RTS window. Awaiting imaging evidence of complete ligamentization would thus delay return by 1–2 years without a demonstrated functional benefit; by analogy with the more extensively studied knee, the degree of imaging maturation does not correlate with clinical outcome or joint laxity [30]. This supports the use of validated criterion‐based tools such as the Ankle‐GO score for RTS decision‐making rather than reliance on graft imaging.

Preoperative isokinetic strength testing was not performed and would have provided an additional objective baseline.

Furthermore, the athlete had a long‐standing history of CLAI and was therefore already familiar with neuromuscular training and proprioceptive strategies, which may have contributed to her rapid postoperative recovery and underline the importance of preventive neuromuscular conditioning in high‐level athletes. Rehabilitation quality may represent a stronger determinant of RTS success than surgical technique alone.

## 4. Conclusion

Arthroscopic anatomic ATFL/CFL reconstruction using gracilis autograft enabled an elite female football player to return to full professional competition and international duty within 12 weeks. This ultrafast RTP was supported by early mechanical stability, comprehensive rehabilitation, and systematic objective monitoring. This case challenges conventional timelines and suggests that, in select high‐performance contexts, accelerated RTP may be achievable without compromising safety.

## Author Contributions

Pierre‐Jean Lambrey: design, writing, and proofreading. Germain Saniel: data collection. Ronny Lopes: design, data collection, and proofreading.

## Funding

No funding was received for this manuscript.

## Ethics Statement

Ethics approval was obtained from the institutional review board (COS‐RGDS‐2025‐10‐002‐LOPES‐R). Written informed consent for publication was obtained from the athlete.

## Conflicts of Interest

The authors declare no conflicts of interest.

## Data Availability

All clinical source documents are available upon reasonable request.

## References

[1] Xu D. , Zhou H. , Yuan Y. , Zhang Z. , Jie T. , Zhou Z. , Gao Z. , Xiang L. , Wang M. , and Gu Y. , Data-Driven Computing Ligament Loading Mechanisms: Integration of the Computational Ligament Mechanics Models With Deep Learning, Acta Mechanica Sinica. (2026) 42, no. 5, 625304, 10.1007/s10409-025-25304-x.

[2] Mustakoski I. , Kurittu E. , Vasankari T. , Brinck T. , Parkkari J. , Heinonen O. , and Leppänen M. , Health Problems in Top-Level Female Football Players: A Four-Season Prospective Study in the Finnish Top Football League, Science and Medicine in Football. (2025) 9, no. 4, 450–466, 10.1080/24733938.2025.2524175, 40642898. 40642898

[3] Kurittu E. , Vasankari T. , Brinck T. , Parkkari J. , Heinonen O. J. , Kannus P. , Hänninen T. , Köhler K. , and Leppänen M. , Injury Incidence and Prevalence in Finnish Top-Level Football—One-Season Prospective Cohort Study, Science and Medicine in Football. (2022) 6, no. 2, 141–147, 10.1080/24733938.2021.1917775, 35475750. 35475750

[4] Flore Z. , Hambly K. , De Coninck K. , and Welsch G. , Time-Loss and Recurrence of Lateral Ligament Ankle Sprains in Male Elite Football: A Systematic Review and Meta-Analysis, Scandinavian Journal of Medicine & Science in Sports. (2022) 32, no. 12, 1690–1709, 10.1111/sms.14217, 35904448. 35904448 PMC9804772

[5] Hertel J. and Corbett R. O. , An Updated Model of Chronic Ankle Instability, Journal of Athletic Training. (2019) 54, no. 6, 572–588, 10.4085/1062-6050-344-18, 31162943. 31162943 PMC6602403

[6] Cottom J. M. , Baker J. S. , and Richardson P. E. , The “All-Inside” Arthroscopic Broström Procedure With Additional Suture Anchor Augmentation: A Prospective Study of 45 Consecutive Patients, Journal of Foot & Ankle Surgery. (2016) 55, no. 6, 1223–1228, 10.1053/j.jfas.2016.07.023, 27638269. 27638269

[7] Messer T. M. , Cummins C. A. , Ahn J. , and Kelikian A. S. , Outcome of the Modified Broström Procedure for Chronic Lateral Ankle Instability Using Suture Anchors, Foot and Ankle International. (2000) 21, no. 12, 996–1003, 10.1177/107110070002101203. 11139039

[8] Hong C. C. and Calder J. , Ability to Return to Sports After Early Lateral Ligament Repair of the Ankle in 147 Elite Athletes, Knee Surgery, Sports Traumatology, Arthroscopy. (2023) 31, no. 10, 4519–4525, 10.1007/s00167-022-07270-2, 36480025. 36480025

[9] Ancelin D. and Philippe C. , Functional and Return-to-Sport Outcomes After Arthroscopic Anatomic Reconstruction of the Lateral Ligaments of the Ankle, Foot and Ankle Surgery. (2025) 31, no. 8, 761–767, 10.1016/j.fas.2025.05.010, 40450480. 40450480

[10] Feng S.-M. , Sun Q.-Q. , Xue C. , Maffulli N. , Oliva F. , and Luo X. , Arthroscopic Lateral Ligament Reconstruction for Isolated Chronic Lateral Ankle Instability Is Associated With Longer Recovery Compared to Arthroscopic Broström Repair and Inferior Extensor Retinaculum Augmentation, Injury. (2025) 56, no. 2, 112082, 10.1016/j.injury.2024.112082, 39700785. 39700785

[11] Bouveau V. , Housset V. , Chasset F. , Bauer T. , and Hardy A. , Return to Sports: Rate and Time After Arthroscopic Surgery for Chronic Lateral Ankle Instability, Orthopaedics & Traumatology: Surgery & Research. (2022) 108, no. 7, 103398, 10.1016/j.otsr.2022.103398, 36084915. 36084915

[12] Rupp M.-C. , Degenhardt H. , Winkler P. W. , Hinz M. , Ehmann Y. J. , Imhoff A. B. , Pogorzelski J. , and Themessl A. , High Return to Sports and Return to Work Rates After Anatomic Lateral Ankle Ligament Reconstruction With Tendon Autograft for Isolated Chronic Lateral Ankle Instability, Knee Surgery, Sports Traumatology, Arthroscopy. (2022) 30, no. 11, 3862–3870, 10.1007/s00167-022-06937-0, 35357531. PMC9568480 35357531

[13] White W. J. , McCollum G. A. , and Calder J. D. F. , Return to Sport Following Acute Lateral Ligament Repair of the Ankle in Professional Athletes, Knee Surgery, Sports Traumatology, Arthroscopy. (2016) 24, no. 4, 1124–1129, 10.1007/s00167-015-3815-1, 26438247. 26438247

[14] Bernardeau A. , Bauer T. , Moussa M. K. , Valentin E. , Lopes R. , and Hardy A. , Return to Sport and Satisfaction After Arthroscopic Anatomic Reconstruction of the Lateral Ligaments of the Ankle in Athletes, Orthopaedics & Traumatology: Surgery & Research. (2025) 111, no. 4, 104221, 10.1016/j.otsr.2025.104221, 40074075. 40074075

[15] Leckie I. , Thomas L. , and Weiler R. , Rehabilitation of a Lateral Ankle Reconstruction in a Male Professional Football Player—A Narrative Case Report, Physical Therapy in Sport. (2023) 62, 32–38, 10.1016/j.ptsp.2023.05.004. 37300971

[16] Su T. , Zhu Y.-C. , Du M.-Z. , Jiang Y. F. , Guo Q. W. , Hu Y. L. , Jiao C. , and Jiang D. , Anatomic Reconstruction Using the Autologous Gracilis Tendon Achieved Less Sprain Recurrence Than the Broström-Gould Procedure but Delayed Recovery in Chronic Lateral Ankle Instability, Knee Surgery, Sports Traumatology, Arthroscopy. (2022) 30, no. 12, 4181–4188, 10.1007/s00167-022-07011-5. 35674772

[17] Fares A. , Picot B. , Lopes R. , Nader F. , Bohu Y. , Meyer A. , Gerometta A. , Grimaud O. , Lefevre N. , Moussa M. K. , and Hardy A. , Indicators of Return to Sports at Preinjury Levels Following Surgery for Chronic Ankle Instability: Comparison of ALR-RSI, AOFAS, and Karlsson Scores, Orthopaedic Journal of Sports Medicine. (2025) 13, no. 1, 23259671241302078, 10.1177/23259671241302078, 39811152. 39811152 PMC11729418

[18] Hardy A. , Freiha K. , Moussa M. K. , Valentin E. , Rauline G. , Alvino K. , Fourchet F. , Picot B. , and Lopes R. , Use of Ankle-GO to Assess and Predict Return to Sport After Lateral Ankle Reconstruction for Chronic Ankle Instability, Orthopaedic Journal of Sports Medicine. (2025) 13, no. 3, 23259671251322903, 10.1177/23259671251322903, 40124190. 40124190 PMC11930476

[19] Sigonney F. , Lopes R. , Bouché P.-A. , Kierszbaum E. , Moslemi A. , Anract P. , Stein A. , and Hardy A. , The Ankle Ligament Reconstruction-Return to sport After Injury (ALR-RSI) Is a Valid and Reproducible Scale to Quantify Psychological Readiness Before Returning to Sport After Ankle Ligament Reconstruction, Knee Surgery, Sports Traumatology, Arthroscopy. (2020) 28, no. 12, 4003–4010, 10.1007/s00167-020-06020-6, 32356045. PMC7669765 32356045

[20] Lopes R. , Decante C. , Geffroy L. , Brulefert K. , and Noailles T. , Arthroscopic Anatomical Reconstruction of the Lateral Ankle Ligaments: A Technical Simplification, Orthopaedics & Traumatology: Surgery & Research. (2016) 102, no. 8, S317–S322, 10.1016/j.otsr.2016.09.003, 27692587. 27692587

[21] Martin R. L. , Irrgang J. J. , Burdett R. G. , Conti S. F. , and Van Swearingen J. M. , Evidence of Validity for the Foot and Ankle Ability Measure (FAAM), Foot and Ankle International. (2005) 26, no. 11, 968–983, 10.1177/107110070502601113. 16309613

[22] Li Y. , Su T. , Hu Y. , Jiao C. , Guo Q. , Jiang Y. , and Jiang D. , Return to Sport After Anatomic Lateral Ankle Stabilization Surgery for Chronic Ankle Instability: A Systematic Review and Meta-Analysis, American Journal of Sports Medicine. (2024) 52, no. 2, 555–566, 10.1177/03635465231170699, 37252803. 37252803

[23] Larkins C. G. , Brady A. W. , Aman Z. S. , Dornan G. J. , Haytmanek C. T. , and Clanton TO , Evaluation of the Intact Anterior Talofibular and Calcaneofibular Ligaments, Injuries, and Repairs With and Without Augmentation: A Biomechanical Robotic Study, American Journal of Sports Medicine. (2021) 49, no. 9, 2432–2438, 10.1177/03635465211018645, 34110933. 34110933

[24] Hou Z.-C. , Su T. , Ao Y.-F. , Hu Y. L. , Jiao C. , Guo Q. W. , Ren S. , Li N. , and Jiang D. , Arthroscopic Modified Broström Procedure Achieves Faster Return to Sports Than Open Procedure for Chronic Ankle Instability, Knee Surgery, Sports Traumatology, Arthroscopy. (2022) 30, no. 10, 3570–3578, 10.1007/s00167-022-06961-0, 35419704. 35419704

[25] Matsui K. , Takao M. , Miyamoto W. , and Matsushita T. , Early Recovery After Arthroscopic Repair Compared to Open Repair of the Anterior Talofibular Ligament for Lateral Instability of the Ankle, Orthopaedic and Trauma Surgery. (2016) 136, no. 1, 93–100, 10.1007/s00402-015-2342-3, 26467354. 26467354

[26] Su T. , Wang A. , Guo Q. , Zhu Y. C. , Jiang Y. F. , Hu Y. L. , Jiao C. , and Jiang D. , Both Open and Arthroscopic All-Inside Anatomic Reconstruction With Autologous Gracilis Tendon Restore Ankle Stability in Patients With Chronic Lateral Ankle Instability, Arthroscopy: The Journal of Arthroscopic & Related Surgery. (2023) 39, no. 4, 1035–1045, 10.1016/j.arthro.2022.11.035, 36631354. 36631354

[27] Cordier G. , Boudahmane S. , Ovigue J. , Michels F. , Araujo Nunes G. , and Dallaudiere B. , MRI Assessment of Tendon Graft After Lateral Ankle Ligament Reconstruction: Does Ligamentization Exist?, American Journal of Sports Medicine. (2024) 52, no. 3, 721–729, 10.1177/03635465231225487, 38343192. 38343192

[28] Bilichtin E. , Rougereau G. , Rollet M. E. , de Rousiers A. , Elkaïm M. , Rousselin B. , Bauer T. , and Hardy A. , MRI Evaluation of ATFL and CFL Ligamentization After Anatomical Surgical Reconstruction With a Hamstring Graft, Foot and Ankle Surgery. (2025) 31, no. 1, 74–78, 10.1016/j.fas.2024.07.003, 39112114. 39112114

[29] Rougereau G. , Langlais T. , Elkaim M. , Bachy M. , Bauer T. , Vialle R. , and Hardy A. , Biomechanical Assessment of Ligament Maturation After Arthroscopic Ligament Reconstruction of the Anterior Talofibular Ligament, Orthopaedics & Traumatology: Surgery & Research. (2025) 111, no. 4, 104162, 10.1016/j.otsr.2025.104162, 39805548. 39805548

[30] Lutz P. M. , Achtnich A. , Schütte V. , Woertler K. , Imhoff A. B. , and Willinger L. , Anterior Cruciate Ligament Autograft Maturation on Sequential Postoperative MRI Is Not Correlated With Clinical Outcome and Anterior Knee Stability, Knee Surgery, Sports Traumatology, Arthroscopy. (2022) 30, no. 10, 3258–3267, 10.1007/s00167-021-06777-4, 34739559. PMC9464175 34739559

